# Ginsenoside Re Attenuates Isoproterenol-Induced Myocardial Injury in Rats

**DOI:** 10.1155/2018/8637134

**Published:** 2018-04-22

**Authors:** Quan-wei Wang, Xiao-feng Yu, Hua-li Xu, Yi-chuan Jiang, Xue-zhong Zhao, Da-yuan Sui

**Affiliations:** ^1^Department of Cardiovascular Medicine, First Hospital, Jilin University, Changchun 130021, China; ^2^Department of Pharmacology, College of Pharmacy, Jilin University, Changchun 130021, China

## Abstract

*Objective. Panax ginseng* is widely used for treatment of cardiovascular disorders in China. Ginsenoside Re is the main chemical component of* Panax ginseng*. This study aimed to investigate the protective effect of Ginsenoside Re on isoproterenol-induced myocardial injury in rats.* Methods.* Male Wistar rats were orally given Ginsenoside Re (5, 20 mg/kg) daily for 7 days. Isoproterenol was subcutaneously injected into the rats for two consecutive days at a dosage of 20 mg/kg/day (on 6th and 7th day). Six hours after the last isoproterenol injection, troponin T level and creatine kinase-MB (CK-MB) activity were assayed. Histopathological examination of heart tissues was performed. The levels of malondialdehyde (MDA) and glutathione (GSH) in heart tissues were measured. The nuclear factor erythroid 2-related factor 2 (Nrf2) content in nucleus and the proteins of glutathione cysteine ligase catalytic subunit (GCLC) and glutathione cysteine ligase modulatory subunit (GCLM) in heart tissues were assayed by western blotting method.* Results.* Treatment with Ginsenoside Re at dose of 5, 20 mg/kg reduced troponin T level and CK-MB activity of rats subjected to isoproterenol. The cardioprotective effect of Ginsenoside Re was further confirmed by histopathological examination which showed that Ginsenoside Re attenuated the necrosis and inflammatory cells infiltration. Ginsenoside Re inhibited the increase of MDA content and the decrease of GSH in heart tissues. Moreover, the Nrf2 content in nucleus and the expressions of GCLC and GCLM were significantly increased in the animals treated with Ginsenoside Re.* Conclusion.* These findings suggested that Ginsenoside Re possesses the property to attenuate isoproterenol-induced myocardial ischemic injury by regulating the antioxidation function in cardiomyocytes.

## 1. Introduction

Myocardial injury is the common presentation of the ischemic heart disease. Ischemic heart disease affects a high proportion of the population in both developed and developing countries. Despite the improved clinical care and the availability of modern medicines, previous report predicted that cardiovascular disease will be a major cause of death worldwide by the year 2020 [1].

Reactive oxygen species (ROS) such as free radicals, singlet oxygens, and peroxides are generated during aerobic metabolism as by-products and are tightly controlled by antioxidants. Excess production of ROS or depletion of antioxidants can lead to a state of oxidative stress that can inflict damage to lipids, proteins, and DNA. ROS generation during ischemia induces a peroxidation of the components of myocardium [2]. Following myocardial ischemia, ROS production is usually increased, which can lead to further damage to the myocardium [3]. Living organisms possess a complicated antioxidant system operating either to eliminate ROS or minimize their negative effects. The first line of cellular defense against oxidative injury in the heart as well as most tissues is antioxidant enzymes [4]. There are also low molecular mass antioxidants including chemically different compounds such as glutathione (GSH), vitamin C and vitamin E. GSH is a tripeptide with multiple functions in living organisms. As a carrier of an active thiol group in the form of a cysteine residue, it acts as an antioxidant either directly by interacting with reactive oxygen or by serving as a cofactor for various antioxidant enzymes [5].

In recent years, Traditional Chinese Medicine (TCM) has been widely used in China, Korea, Japan, and other Asian countries in the treatment of cardiovascular diseases. Furthermore, an increasing number of studies have confirmed the efficacy of TCM for treating ischemic heart disease [6].* Panax ginseng* is a perennial herb which belongs to the Araliaceae family and is distributed in 35 countries, particularly in China and South Korea.* Panax ginseng* has been considered as a traditional medicine in Asian countries for thousands of years for its health promotion in various disease conditions.* Panax ginseng* has various effects, such as antifatigue, antiaging, improving immune function [7]. It is also reported that* Panax ginseng *has cardioprotective and neuroprotective properties [8]. Among the components of* Panax ginseng*, ginsenosides have been shown to be the major pharmacological ingredient in* Panax ginseng*. Since the first isolation of six ginsenosides from* Panax ginseng* in the 1960s, plenty of ginsenosides have been isolated and identified from the* Panax ginseng*. Depending on their structures, ginsenosides are divided into three groups: the panaxatriol group including Re, Rf, Rg1, Rg2, and Rh1, the panaxadiol group including Rb1, Rb2, Rb3, Rc, Rd, Rg3, and Rh2, and the oleanolic acid group [9]. This study aimed to evaluate the effect of Ginsenoside Re on myocardial ischemic injury in rats.

## 2. Materials and Methods

### 2.1. Animals

The experiments were performed according to the National Institutes of Health Guidelines for the Care and Use of Laboratory Animals (publication 86–23, revised in 1986) and were approved by the Ethics Committee of Jilin University. Wistar rats weighing 240 to 270 g were provided by the Experiment Animal Center of Norman Bethune University of Medical Science (Jilin, China). The animals were housed in diurnal lighting conditions (12 h/12 h) and allowed free access to food and water for 7 days before the experiment.

### 2.2. Preparation of Extract from* Panax ginseng*

**Figure 1:**
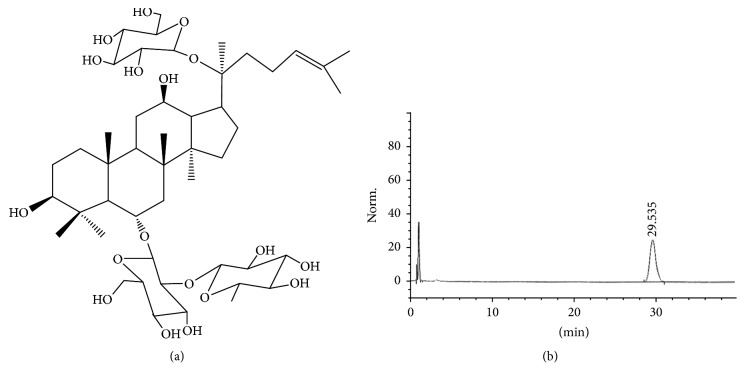
(a) The chemical structure of Ginsenoside Re; (b) HPLC chromatogram of Ginsenoside Re; the peak at 29.535 min was identified as Ginsenoside Re.

Samples of* Panax ginseng* were collected from Tonghua country of Changchun city in Jilin Province during June 2015. Its identity was confirmed by Professor Yanping Chen with authentic specimens retained at the herbarium, Jilin University. A voucher specimen was deposited in Department of Medicinal Chemistry, Jilin University.* Panax ginseng* (5 kg) was pounded into powder and then was soaked with 95% ethanol (1 : 2, w : w) for 12 h. After being filtrated, the extracts were added to nonpolar macroporous resin (D4020 Tianjin, China) column and the impurities were washed out by water. Then total saponin was eluted by 75% ethanol. The eluent was freeze-dried finally giving amorphous powder. The residue was dissolved with 75% ethanol again and the suspension was filtrated. The precipitate was subjected to a silica gel column chromatography (2 cm × 20 cm) and eluted with a stepwise gradient of trichloromethane : methanol : acetic ether : H_2_O (15 : 22 : 40 : 23) and then underwent further chromatography on silica gel columns, employing the same eluent systems, to give a Ginsenoside Re. The yield of the extract as a dried material was approximately 0.45% by weight of the original material. Further analysis by HPLC showed that the content of Ginsenoside Re in the resultant extract was 98.6% (Figure 1).

### 2.3. Chemical Reagents

The ELISA kit of troponin T was purchased from Westang Biomedical Technology Company (Shanghai, China). CK-MB kit was obtained from Biosino Biotechnology Company Ltd. (Beijing, China). Isoproterenol and GSH were purchased from Sigma Chemical Company (St. Louis, MO, USA). Carboxymethyl cellulose sodium salt (CMC-Na) was from Shanghai Jinshan Chemical Co., Ltd. (Shanghai, China). Other reagents were of commercially analytical grade.

### 2.4. Experimental Protocols

**Figure 2:**
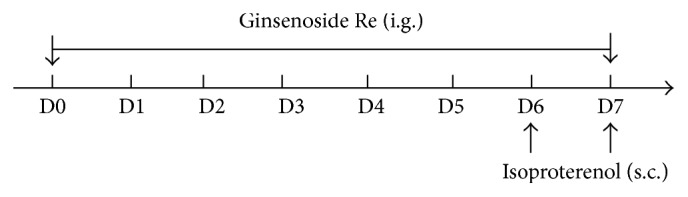
Experimental design.

Fifty-six rats were randomly divided into four groups with 14 animals in each group: control, isoproterenol, and Ginsenoside Re (5 and 20 mg/kg) groups. Rats in control and isoproterenol groups were orally given 0.5% CMC-Na daily for 7 days. While animals in Ginsenoside Re groups were intragastrically treated with Ginsenoside Re at doses of 5 or 20 mg/kg, respectively, daily for 7 days. Isoproterenol (20 mg/kg) was dissolved in normal saline and subcutaneously injected to rats at an interval of 24 h for 2 days (on 6th and 7th day) to induce myocardial ischemic injury [10]. Isoproterenol injection was performed at 2 h after Ginsenoside Re treatment (Figure 2).

### 2.5. Assay of Troponin T Level and CK-MB Activity in Serum

The animals were anaesthetized with isoflurane and blood samples were collected in dry test tubes without anticoagulant at 24 h after the last isoproterenol injection. The samples were centrifuged (3500* g*) at 4°C for 10 min. Then the serum was harvested and stored at −80°C for biochemical assay. According to the manufacturer's instructions, troponin T level was assayed using an ELISA kit. The activity of CK-MB was analyzed using commercial kits by employing an automatic biochemical analyzer (COBAS-FARA, Germany).

### 2.6. Histopathological Examination

Three heart tissues obtained from all groups were washed with normal saline and then fixed in phosphate buffered 10% formalin solution. After fixation, heart tissues were embedded in paraffin and then were cut into 5 *μ*m thick sections. Stained with hematoxylin-eosin, the sections were examined by an experienced observer who was unaware of the animal experimental groups under light microscope and then photomicrographs were taken.

### 2.7. MDA and GSH Assays

After blood collection, eight animals of each group were sacrificed by decapitation. The hearts were homogenized in 4 volumes of 0.1 mol/L ice phosphate buffer (pH 7.4) and then were centrifuged at 10,000* g* for 10 min at 4°C. The total protein in the supernatant was estimated by BCA protein assay kit (Beyotime Institute of Biotechnology, Shanghai, China). The MDA content in heart tissues was assayed according to the previous method [11]. GSH was measured employing o-phthalaldehyde condensation reaction with GSH at pH 8.0. Readings were taken at activation/emission wave lengths of 340/420 nm [12].

### 2.8. Western Blot

The nuclear and cytoplasmic protein extracts were prepared using nuclear and cytoplasmic protein extraction kit (heart tissues of three rats in each group). Proteins were loaded (60 *μ*g) and subjected to 10% sodium dodecyl sulfate-polyacrylamide gel electrophoresis. Proteins were transferred to polyvinylidene fluoride membranes (Millipore Corporation) for 1.5 h at 100 V. Blocked with 5% nonfat milk for 1 h, the membranes were incubated with the primary antibodies: rabbit anti-Nrf2 (1 : 1500, Abcam), anti-GCLC (1 : 1500, Abcam), anti-GCLM (1 : 1000, Abcam), anti-Lamin B1 (0.1 *μ*g/ml, Abcam), or rabbit anti-*β*-Actin (1 : 1500; Cell Signaling) overnight at 4°C. The membranes were processed with the respective horseradish peroxidase-labeled secondary antibody (1 : 3000). Bands were visualized using the ECL detection reagents (Beyotime Institute of Biotechnology, Shanghai, China).

### 2.9. Statistical Analysis

All data were expressed as the mean ± SD. Analysis was performed using SPSS 22.0. The data were analyzed with one-way ANOVA followed by Tukey post hoc test. Statistical significance was defined as *P* < 0.05.

## 3. Results

### 3.1. Effect of Ginsenoside Re on the Troponin T Level in Myocardial Ischemic Rats

**Figure 3:**
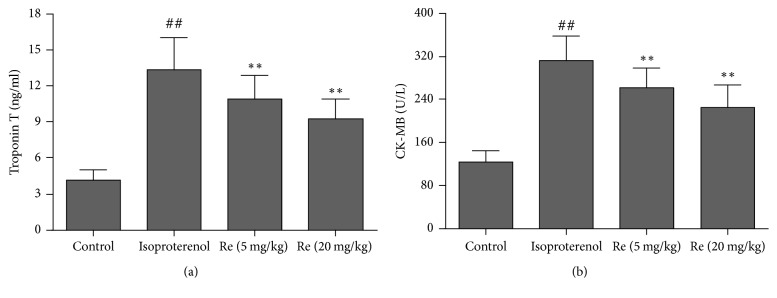
Effect of Ginsenoside Re on the troponin T level (a) and CK-MB activity (b) in myocardial ischemic rats. Data were expressed as the mean ± SD (*n* = 14). ^##^*P* < 0.01 compared with the control group; ^*∗∗*^*P* < 0.01 compared with the isoproterenol group.

Compared with the control group (4.2 ± 0.82 ng/ml), the troponin T level was significantly increased in isoproterenol group (13.3 ± 2.68 ng/ml, *P* < 0.01). Compared with the isoproterenol group, the troponin T level in the Ginsenoside Re (5 and 20 mg/kg) groups was significantly reduced (10.8 ± 2.00 ng/ml, 9.3 ± 1.63 ng/ml, resp., *P* < 0.01), (Figure 3(a)).

### 3.2. Effect of Ginsenoside Re on the CK-MB Activity in Myocardial Ischemic Rats

As shown in Figure 3(b), there was a significant increase in the CK-MB activity observed in the isoproterenol group (*P* < 0.01). Compared with isoproterenol group, treatment with Ginsenoside Re at doses of 5 and 20 mg/kg significantly inhibited the augment of the CK-MB activity in isoproterenol-induced myocardial ischemic rats (*P* < 0.01).

### 3.3. Effect of Ginsenoside Re on Pathological Changes in Myocardium

**Figure 4:**
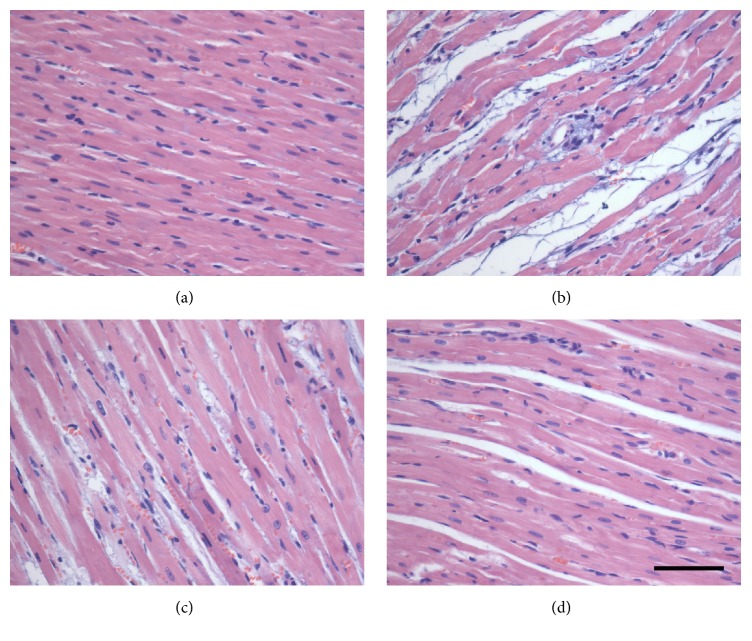
Effects of Ginsenoside Re on pathological changes of heart tissues in isoproterenol-induced myocardial ischemic rats (H&E). (a) Control group; (b) isoproterenol group; (c) Ginsenoside Re at dose of 5 mg/kg group; (d) Ginsenoside Re at dose of 20 mg/kg group. Bar = 400 *μ*m.

In control group, myocardium showed clear integrity of myocardial cell membrane and no inflammatory cells infiltration was observed. Heart tissues from isoproterenol-induced myocardial injury rats showed widespread myocardial structure disorder and subendocardial necrosis, which was related to inflammatory cells infiltration and myofibrillar fracture. The rats in Ginsenoside Re (5 mg/kg) group exhibited mild necrosis and less infiltration of inflammatory cells. The heart tissues of animals in Ginsenoside Re at dose of 20 mg/kg showed nearly normal myofibrillar structure with few neutrophil granulocytes infiltration (Figure 4).

### 3.4. Effect of Ginsenoside Re on the MDA Content in Myocardial Ischemic Rats

**Figure 5:**
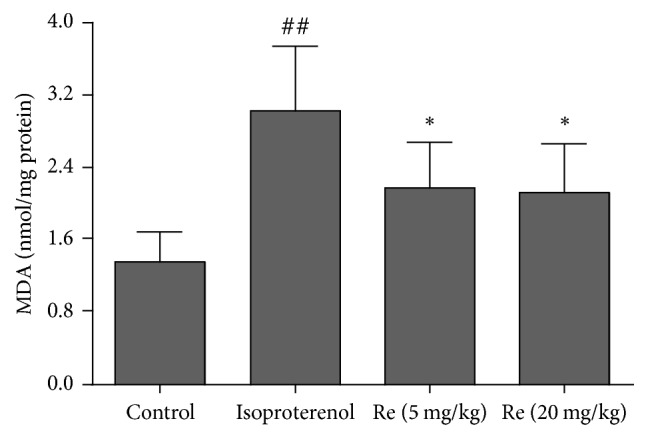
Effect of Ginsenoside Re on the MDA content level in myocardial ischemic rats. Data were expressed as the mean ± SD (*n* = 8). ^##^*P* < 0.01 compared with the control group; ^*∗*^*P* < 0.05 compared with the isoproterenol group.

The lipid peroxidation end product MDA was measured in heart tissues of rats. Compared with the control group (1.34 ± 0.34 nmol/mg protein), MDA content was significantly augmented after isoproterenol injection (3.03 ± 0.72 nmol/mg protein, *P* < 0.01). Treatment with Ginsenoside Re at a doses of 5, 20 mg/kg attenuated the increase of MDA content (2.17 ± 0.50, 2.12 ± 0.54 nmol/mg protein, resp., *P* < 0.05, Figure 5).

### 3.5. Effect of Ginsenoside Re on the GSH Level in Myocardial Ischemic Rats

**Figure 6:**
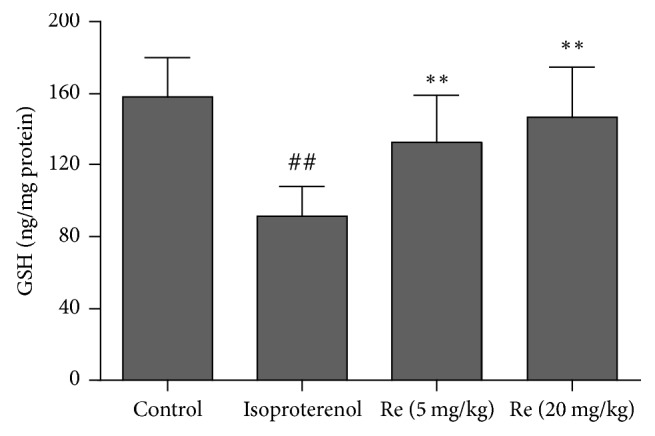
Effect of Ginsenoside Re on the GSH level in myocardial ischemic rats. Data were expressed as the mean ± SD (*n* = 8). ^##^*P* < 0.01 compared with the control group; ^*∗∗*^*P* < 0.01 compared with the isoproterenol group.

In the control group, the level of GSH in the heart tissues was 158.2 ± 21.3 ng/mg protein. There was a significant decrease of GSH level (91.8 ± 16.2 ng/mg protein) observed in isoproterenol-induced myocardial ischemic rats (*P* < 0.01) as compared to the control group. Compared with the isoproterenol group, the Ginsenoside Re (5, 20 mg/kg) groups showed a significant increase of GSH level (132.6 ± 26.7, 146.4 ± 27.9 ng/mg protein, resp.) in the heart tissues (*P* < 0.01, Figure 6).

### 3.6. Effect of Ginsenoside Re on the Nrf2 Content in Myocardial Ischemic Rats

**Figure 7:**
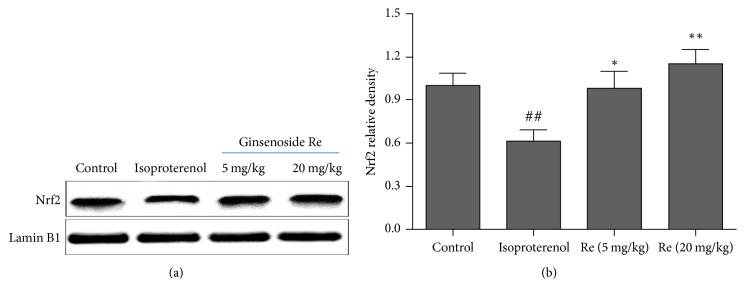
Effect of Ginsenoside Re on the Nrf2 content in myocardial ischemic rats. (a) shows representative photographs of Nrf2 in western blotting measurement; (b) indicates bar graphs of Nrf2 content in western blotting measurement. Data were expressed as the mean ± SD (*n* = 3). ^##^*P* < 0.01 compared with the control group; ^*∗*^*P* < 0.05, ^*∗∗*^*P* < 0.01 compared with the isoproterenol group.

The Nrf2 content in nucleus of cardiomyocytes was assayed by western blot method. Compared to the control group, the Nrf2 content was markedly decreased in isoproterenol group (*P* < 0.01). Ginsenoside Re treatment increased the Nrf2 content in nucleus of cardiomyocytes (*P* < 0.05 or *P* < 0.01; Figure 7).

### 3.7. Effect of Ginsenoside Re on the Expressions of GCLC and GCLM in Myocardial Ischemic Rats

**Figure 8:**
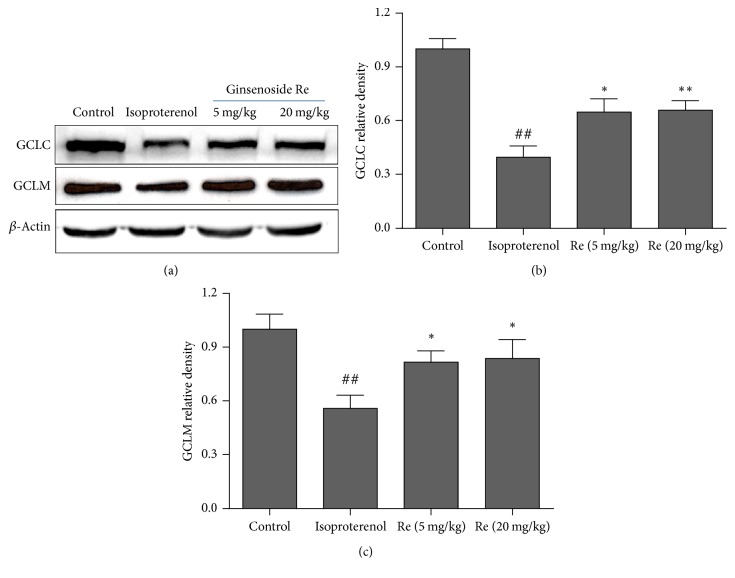
Effect of Ginsenoside Re on the expressions of GCLC and GCLM in myocardial ischemic rats. (a) shows representative photographs of GCLC and GCLM in western blotting measurement; (b) indicates bar graphs of GCLC and GCLM expressions in western blotting measurement. ^##^*P* < 0.01 compared with the control group; ^*∗*^*P* < 0.05, ^*∗∗*^*P* < 0.01 compared with the isoproterenol group.

Figure 8 showed the effect of Ginsenoside Re on the expressions of GCLC and GCLM in isoproterenol-induced myocardial ischemic rats. The expressions of GCLC and GCLM in the isoproterenol group decreased markedly as compared with that of the control group (*P* < 0.01). However, after treatment with Ginsenoside Re at doses of 5, 20 mg/kg, the expressions of GCLC and GCLM increased significantly (*P* < 0.05 or *P* < 0.01).

## 4. Discussion

Isoproterenol is a *β*-adrenergic agonist. It has acute positive inotropic and chronotropic effects on the heart. The strong inotropic and chronotropic actions of isoproterenol will induce necrosis, inflammatory cell infiltration, hypertrophy, and fibrosis [13]. Histological analysis has shown that pathophysiological and morphological aberrations produced in the heart of rats with isoproterenol are comparable with those of ischemic heart disease patients. Up to date, isoproterenol-induced myocardial ischemic injury in animals is widely used to evaluate the effects of cardioprotective agents. The findings of this study showed that Ginsenoside Re reduced the troponin T level and CK-MB activity in myocardial ischemic rats, which suggested that Ginsenoside Re possessed cardioprotective effect. Based on the Ginsenoside Re-induced increase of Nrf2 and GSH level, it indicated that the mechanisms of its action were associated with regulating the antioxidation function in cardiomyocytes.

Troponin T, a regulatory protein controlling the calcium-mediated interaction of actin and myosin contractile protein, is a highly specific and sensitive marker in the diagnosis of human and animal model of myocardial ischemic injury [14]. Elevation of serum troponin T level predicts the risk of both myocardial ischemic injury and subsequent cardiac death. CK-MB is present in cardiomyocytes. After myocardial ischemia, CK-MB will leak out from the damaged tissues to the blood stream when the myocardium membrane becomes permeable or rupture. Therefore, troponin T and CK-MB in blood are highly useful in detecting myocardial ischemic injury. This study showed that isoproterenol injection led to a significant increase in troponin T level and CK-MB activity. The histopathological examination found that isoproterenol induced myocardium necrosis, inflammatory cells infiltration, and separation of cardiac muscle fibers, whereas treatment with Ginsenoside Re decreased the troponin T level and CK-MB activity in serum. Ginsenoside Re also attenuated the pathological changes induced by isoproterenol administration. These findings indicated that Ginsenoside Re possessed cardioprotective effects by preventing the cardiomyocytes damage thereby restricting the leakage of troponin T and CK-MB from the myocardium into the blood.

Oxidative stress is well documented in the pathogenesis of humans as well as isoproterenol-induced myocardial ischemic injury. In rats, isoproterenol had been reported to increase oxygen demand, deplete ATP levels, cause calcium overload, and undergo autoxidation leading to formation of free radicals [15]. Cardiomyocyte membrane contains huge quantity of polyunsaturated fatty acids in its phospholipids. MDA is a major lipid peroxidation end product of cardiomyocyte membrane oxidation. The increase of MDA content indicates the oxidative stress which results in an irreversible damage to heart tissues of animals subjected to isoproterenol administration [16]. GSH is a principal low molecular weight thiol antioxidant. The synthesis of GSH from its constituent amino acids involves the actions of two enzymes, glutamate-cysteine ligase (GCL) and GSH synthetase. GCL, the rate-controlling enzyme in the pathway of GSH synthesis, is a heterodimer composed of a GCLC and a GCLM. GCLM modulates the catalytic properties of GCLC by lowering its sensitivity to the inhibition of GSH and by increasing its affinity to glutamate. Without the presence of GCLM, GCLC would function poorly in vivo [17]. The basal and inducible expression of GCLC and GCLM is mediated by means of the antioxidant response element (ARE). The ARE is an enhancer sequence that transcriptionally regulates antioxidant enzymes, which are critical for maintaining cellular redox status and protecting against oxidative damage [18]. It shows that Nrf2 is the principal transcription factor which regulates ARE-mediated gene transcription [19]. Under conditions of oxidative stress, Nrf2 is translocated to the nucleus to upregulate genes involved in antioxidant defense. Nrf2 is the main transcription factor required for the activation of both GCLC and GCLM genes in humans via binding with the AREs. In response to exposure of various oxidants, Nrf2 binds to AREs presenting in the human GCLC and GCLM promoters [20]. In human monocytes, the tumor necrosis factor alpha has been shown to mediate the sustained activation of Nrf2, leading to induction of GCLM expression [21]. In rodent models, the Nrf2 transcription factor has been demonstrated to be involved in the basal induction of GCLM. It showed that Nrf2-knockout mice displayed a significantly lower GSH level and a reduced basal expression of the GCLM [22]. Previous study also reported that there was a reduced basal expression of GCL observed in Nrf2-null mice [23]. In this study, we observed that Ginsenoside Re resulted in an increase of Nrf2 nuclear translocation in isoproterenol-induced myocardial injury rats. Moreover, our data showed that the expressions of GCLC and GCLM were enhanced after Ginsenoside Re treatment. In line with these findings, Ginsenoside Re also led to the increase of GSH content followed by the decrease of MDA level. These results suggested that the cardioprotective effects of Ginsenoside Re are attributed to the antioxidative properties by augmenting Nrf2-induced expression of GCLC and GCLM, at least, in part.

## 5. Conclusions

In summary, this study indicated that Ginsenoside Re possessed cardioprotective effects in isoproterenol-induced myocardial injury rats. The pharmacological action of Ginsenoside Re was related to regulating the antioxidation function in cardiomyocytes.

## References

[1] Murray C. J. L., Lopez A. D. (1997). Alternative projections of mortality and disability by cause 1990–2020: global burden of disease study. *The Lancet*.

[2] Lesnefsky E. J., Slabe T. J., Stoll M. S. K., Minkler P. E., Hoppel C. L. (2001). Myocardial ischemia selectively depletes cardiolipin in rabbit heart subsarcolemmal mitochondria. *American Journal of Physiology-Heart and Circulatory Physiology*.

[3] Siddiqui T., Zia M. K., Ali S. S., Rehman A. A., Ahsan H., Khan F. H. (2016). Reactive oxygen species and anti-proteinases. *Archives of Physiology and Biochemistry*.

[4] Papaharalambus C. A., Griendling K. K. (2007). Basic mechanisms of oxidative stress and reactive oxygen species in cardiovascular injury. *Trends in Cardiovascular Medicine*.

[5] Lushchak V. I. (2012). Glutathione homeostasis and functions: potential targets for medical interventions. *Journal of Amino Acids*.

[6] Duan L., Xiong X., Hu J., Liu Y., Li J., Wang J. (2017). Panax notoginseng saponins for treating coronary artery disease: A functional and mechanistic overview. *Frontiers in Pharmacology*.

[7] Chu S.-F., Zhang J.-T. (2009). New achievements in ginseng research and its future prospects. *Chinese Journal of Integrative Medicine*.

[8] Xu W., Choi H.-K., Huang L. (2017). State of Panax ginseng research: A global analysis. *Molecules*.

[9] Lee Y.-M., Yoon H., Park H.-M., Song B. C., Yeum K.-J. (2017). Implications of red Panax ginseng in oxidative stress associated chronic diseases. *Journal of Ginseng Research*.

[10] Gai Y., Ma Z., Yu X., Qu S., Sui D. (2012). Effect of ginsenoside Rh1 on myocardial injury and heart function in isoproterenol-induced cardiotoxicity in rats. *Toxicology Mechanisms and Methods*.

[11] Ohkawa H., Ohishi N., Yagi K. (1979). Assay for lipid peroxides in animal tissues by thiobarbituric acid reaction. *Analytical Biochemistry*.

[12] Cohn V. H., Lyle J. (1966). A fluorometric assay for glutathione. *Analytical Biochemistry*.

[13] Tasatargil A., Kuscu N., Dalaklioglu S. (2017). Cardioprotective effect of nesfatin-1 against isoproterenol-induced myocardial infarction in rats: Role of the Akt/GSK-3*β* pathway. *Peptides*.

[14] Ahmed R., Tanvir E. M., Hossen M. S. (2017). Antioxidant properties and cardioprotective mechanism of Malaysian propolis in rats. *Evidence-Based Complementary and Alternative Medicine*.

[15] Padmanabhan M., Prince P. S. M. (2006). Preventive effect of S-allylcysteine on lipid peroxides and antioxidants in normal and isoproterenol-induced cardiotoxicity in rats: A histopathological study. *Toxicology*.

[16] Sharmila Queenthy S., Stanely Mainzen Prince P., John B. (2017). Diosmin Prevents Isoproterenol-Induced Heart Mitochondrial Oxidative Stress in Rats. *Cardiovascular Toxicology*.

[17] Fraser J. A., Kansagra P., Kotecki C., Saunders R. D. C., McLellan L. I. (2003). The Modifier Subunit of Drosophila Glutamate-Cysteine Ligase Regulates Catalytic Activity by Covalent and Noncovalent Interactions and Influences Glutathione Homeostasis in Vivo. *The Journal of Biological Chemistry*.

[18] Lu S. C. (2009). Regulation of glutathione synthesis. *Molecular Aspects of Medicine*.

[19] Suh J. H., Shenvi S. V., Dixon B. M. (2004). Decline in transcriptional activity of Nrf2 causes age-related loss of glutathione synthesis, which is reversible with lipoic acid. *Proceedings of the National Acadamy of Sciences of the United States of America*.

[20] Levonen A.-L., Landar A., Ramachandran A. (2004). Cellular mechanisms of redox cell signalling: Role of cysteine modification in controlling antioxidant defences in response to electrophilic lipid oxidation products. *Biochemical Journal*.

[21] Rushworth S. A., Shah S., MacEwan D. J. (2011). TNF mediates the sustained activation of Nrf2 in human monocytes. *The Journal of Immunology*.

[22] Yang H., Magilnick N., Lee C. (2005). Nrf1 and Nrf2 regulate rat glutamate-cysteine ligase catalytic subunit transcription indirectly via NF-*κ*B and AP-1. *Molecular and Cellular Biology*.

[23] Chan K., Han X.-D., Kan Y. W. (2001). An important function of Nrf2 in combating oxidative stress: detoxification of acetaminophen. *Proceedings of the National Acadamy of Sciences of the United States of America*.

